# Current Approaches to Microplastics Detection and Plastic Biodegradation

**DOI:** 10.3390/molecules30112462

**Published:** 2025-06-04

**Authors:** Paula Przygoda-Kuś, Katarzyna E. Kosiorowska, Aneta K. Urbanek, Aleksandra M. Mirończuk

**Affiliations:** 1 Institute of Biology, Laboratory for Biosustainability, Wrocław University of Environmental and Life Sciences, 51-631 Wrocław, Poland; paula.przygoda-kus@upwr.edu.pl (P.P.-K.); katarzyna.kosiorowska@upwr.edu.pl (K.E.K.); aneta.urbanek@upwr.edu.pl (A.K.U.); 2 Department of Applied Bioeconomy, Wroclaw University of Environmental and Life Sciences, 51-630 Wrocław, Poland

**Keywords:** biodegradation, methods, plastic biodegradation, synthetic polymers

## Abstract

Environmental concerns about the widespread use of non-biodegradable plastic have generated interest in developing quick and effective methods to degrade synthetic polymers. With millions of tons of plastic waste generated annually, biodegradation by microorganisms presents a promising and eco-friendly solution. However, a bottleneck has arisen due to the lack of standardized methods for verification of the biodegradation process. Based on this literature review, he techniques most commonly employed for this purpose currently include measuring mass loss, examining the surface of plastic fragments by scanning electron microscopy (SEM) and atomic force microscopy (AFM), and using analytical methods such as Fourier transform infrared spectroscopy (FTIR), pyrolysis–gas chromatography–mass spectrometry (Pyr-GC/MS) or high-performance liquid chromatography (HPLC). Each of these methods has its advantages and disadvantages. Nevertheless, currently, there is no universal approach to accurately assess the ability of individual microorganisms to degrade plastics. In this review, we summarize the latest advances in techniques for detecting biodegradation of synthetic polymers and future directions in the development of sustainable strategies for mitigating plastic pollution.

## 1. Introduction

Recently, synthetic polymers have become an irreplaceable element in everyday life due to their low cost, durability, and versatility, which have made them an attractive substitute material for metal, glass, wood, or paper. Plastics are used in almost every aspect of our lives, from packaging, textile production, and personal care products to construction applications [1]. The global production of plastic has been growing rapidly since 1950. Due to the COVID-19 pandemic, there was a short stagnation in plastic production. However, in 2023, synthetic polymers production reached 413.8 Mt, which is the highest amount recorded so far [2,3]. Approximately 80% of the total global plastic consumption is made up of petrochemical plastics, including polyethylene terephthalate (PET), polyethylene (PE), polypropylene (PP), polystyrene (PS), and polyvinyl chloride (PVC) [4,5,6].

Plastic waste due to its difficult biodegradation and high volume of production is now regarded as a global environmental pollutant. The majority of produced plastics are described as non-biodegradable, since the process of their decomposition is slow in the natural environment, such that it cannot be detected during a human lifetime. Until the last decade, it was believed that petrochemical polymers were not susceptible to biological decomposition; however, there is a rising number of scientific papers on microorganisms capable of degrading plastics [7,8]. Nonetheless, the degradation of synthetic plastics remains challenging due to their high molecular weight, dense C-C skeleton, hardness, and insolubility in water as well as the addition of antioxidants and stabilizers during synthesis. Moreover, long-chain polymers cannot be carried across microbial cell membranes, which limits their accessibility to microorganisms [9,10]. To allow penetration into the microbial cells, long-chain polymers must initially be depolymerized into short-chain or low-molecular-weight products. These products can then pass through cell membranes and be assimilated during intracellular metabolic pathways. Also worth mentioning is that the vulnerability to biological decomposition varies based on the chemical and physical characteristics, such as morphology (amorphous forms degrade faster than crystalline), physical form (fibers, films, pellets or powder), and the presence of easily breakable bonds such as ester or amide bonds.

The fragmentation of plastics into smaller particles, known as microplastics (MPs), further increases environmental concerns; environmental ageing can release additives and increase the toxicity of MPs [11]. MPs are classified based on size, shape, origin, and composition. Plastic particles based on size are categorized as macroplastic, for particles over 25 mm, mesoplastic, for particles between 5 and 25 mm, large microplastic, 1–5 mm, small microplastic, 1 µm−1 mm, and nanoplastic, for particles measuring < 1 µm. Based on shape, MPs are classified into granules, fragments, films, pellets, fibers, and foams. MPs consist of different types of polymers, but the most common are listed above [3,5,6]. A similar classification is also made based on their origin, i.e., into primary and secondary MPs. MPs can be produced intentionally (they are used, for example, in the production of cosmetics, such as scrubs and toothpaste), in which case they are called primary MPs. They can also be generated indirectly through processes such as littering, abrasion, irrigation, mulching, and the use of sewage sludge and compost in agriculture, in which case they are classified as secondary MPs [3,12].

Not only are MPs only environmental pollutants, but they also pose risks to human and animal health. Detecting MPs is therefore essential for understanding their environmental distribution, ecological impacts, and pathways into biological systems. Advanced analytical methods, including spectroscopy, microscopy, and chromatography, have been utilized to identify and quantify MPs, providing critical insights for developing effective mitigation strategies. Research indicates that MPs can be ingested by aquatic organisms such as mussels, fish, and marine mammals, and that they can bioaccumulate through the food chain, ultimately entering the human diet [13,14,15]. MPs have been detected in human feces [16] and shown to penetrate organs such as the liver, intestines, and lungs in animal models, as well as cross the blood–brain barrier, raising concerns about their neurotoxicity [17]. Behavioral studies highlight the impact of MPs on organisms. For example, exposure to PE particles caused atypical behavior in *Danio rerio* fish, including twitching and unnatural tail positioning, with severity increasing with higher MP concentrations [18,19].

Due to the environmental effects of the excessive use of non-biodegradable plastics, more and more biodegradable plastics are being invented, which offers a promising approach to managing the problem of plastic waste accumulation in the oceans and soil. These polymers can be degraded faster than “conventional” plastics when exposed to light, oxygen, moisture and UV radiation. They can be degraded by microorganisms into small molecules such as water, carbon dioxide, methane, etc. [1]. There are several different types of biodegradable plastics, e.g., polylactic acid (PLA), polybutylene succinate (PBS), polyhydroxyalkanoates (PHA), and polycaprolactone (PCL) [4]. Despite this progress, a comprehensive solution to plastic waste accumulation is yet to be found.

To solve the global problem of plastic waste, researchers have focused on the isolation and identification of microorganisms with the capability of plastic biodegradation. Many natural and engineered strains have shown potential in this process [8,20,21]. However, due to the diversity in the chemical structure of polymers, studies to identify novel microorganisms and enzymes are still ongoing.

Developing a universal method for detecting MPs and assessing their degradation is crucial for addressing the growing environmental and health impacts of plastic pollution. Current approaches often require specialized techniques tailored to specific MPs types, making cross-comparisons and global assessments challenging. A standardized detection method would not only facilitate accurate quantification and classification of MPs across different environments but also support the evaluation of biodegradation processes by providing consistent metrics to measure microbial or enzymatic efficiency. This review aims to summarize the recent advances in MPs detection technologies while highlighting promising strategies for evaluating the biodegradation of this persistent micropollutant. Additionally, it seeks to identify critical research gaps that must be addressed to develop holistic solutions for effective evaluation of plastic biodegradation.

## 2. Analytical Techniques Used to Detect Biodegradation of Plastics

**Figure 1:**
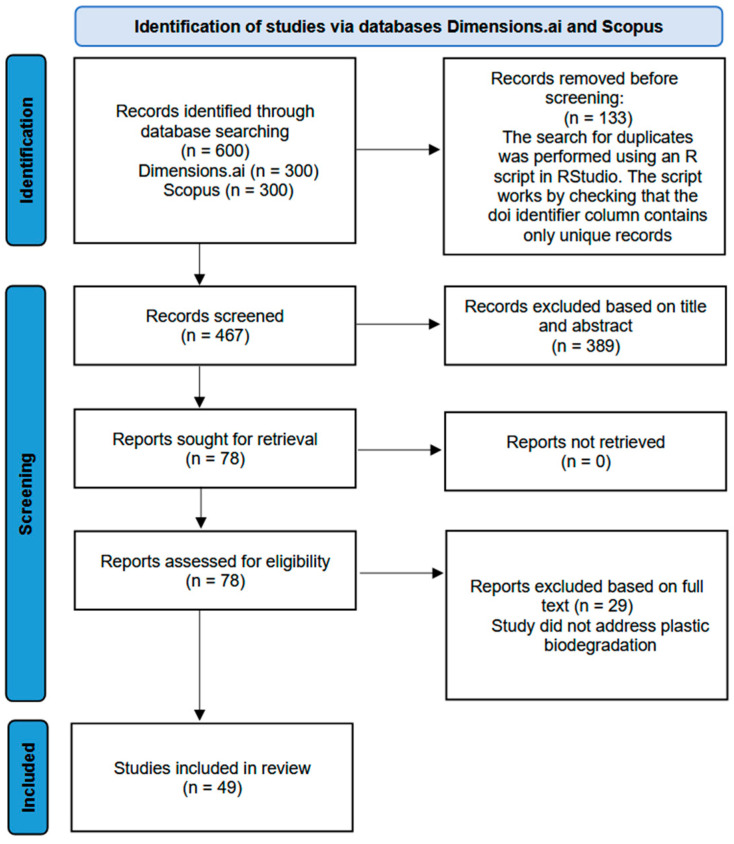
Diagram representation of structured literature search (graph adapted from [22]).

**Figure 2:**
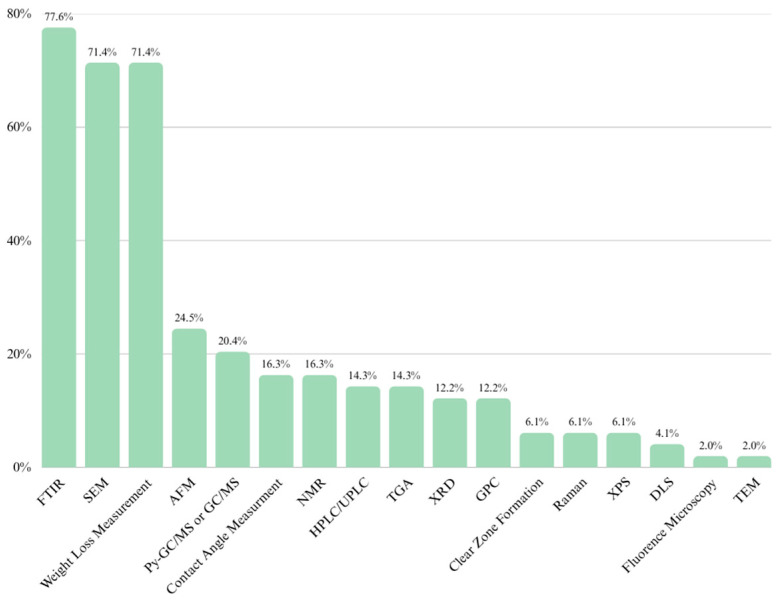
Commonly used methods for detecting biodegradation based on the structured literature search [graph created with the free version of canva.com].

The methodology used to identify predominant techniques for confirming plastic biodegradation involved a structured literature search conducted on the Scopus and Dimensions.ai databases. Only Open Access articles published in English were considered. Specific keywords, including “microplastic biodegradation organism”, “microplastic biodegradation microbes”, and “plastic biodegradation” were employed for the search. Results were filtered by citation metrics and relevance. From each query, 50 results were retrieved, resulting in an initial dataset of 600 articles. Duplicate entries were removed, reducing the dataset to 467 articles. Titles and abstracts were then reviewed in the first stage of selection, narrowing the dataset to 78 articles. After a detailed review of the full texts, the methods used for confirming biodegradation were extracted from 49 articles to determine the most frequently employed techniques. Figure 1 presents a workflow applied to retrieve articles relevant to the determination of the most frequently used techniques to confirm biodegradation. Figure 2 presents the distribution of the identified techniques based on their frequency of use in the studied articles.

**Figure 3:**
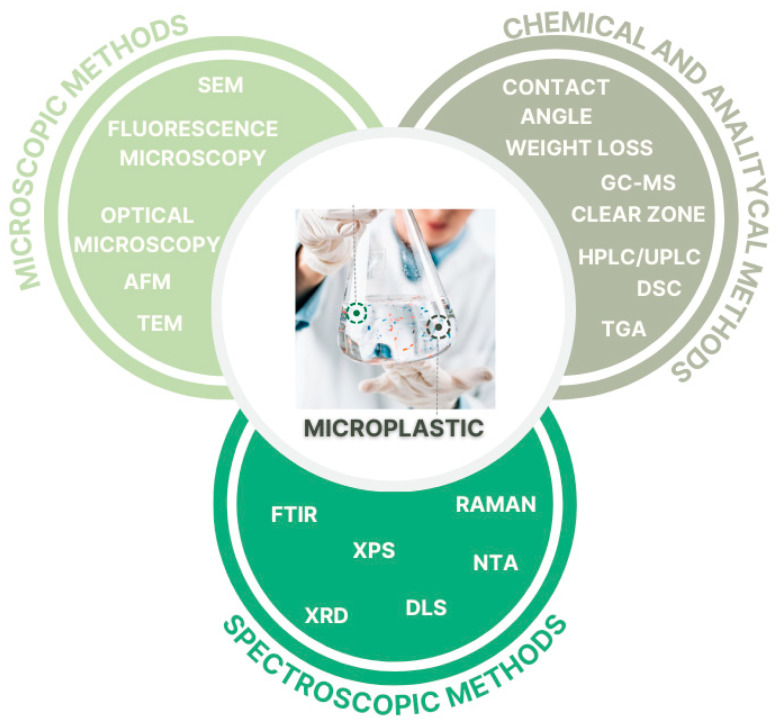
Commonly applicable methods for detecting and characterizing MPs [graph created with the free version of canva.com].

In the search for efficient methods for biodegradation of plastics, it is crucial to establish rapid, reliable, and standardized methods for validating the occurrence of the biodegradation process. The techniques identified through this literature review highlight the importance of various microscopic, spectroscopic, chemical, and analytical methods in detecting and characterizing MPs and their biodegradation. These approaches are summarized in Figure 3.

The following sections of this article will study five of the most commonly used methods presented in Figure 2, along with additional relevant techniques. Their advantages, limitations, and prospects for application in biodegradation studies will also be analyzed to provide a comprehensive understanding of their roles in confirming the biodegradation process of synthetic polymers.

### 2.1. Microscopic Methods

#### 2.1.1. Scanning Electron Microscopy

Observation of the plastic film surface is one of the approaches to confirm the biodegradation process. SEM relies on the principle of examining morphological changes, such as surface irregularities, the formation of various pores and pits, and development of cracks. It provides detailed insight into changes occurring on the surface of the material due to microbial activity. It provides images with high resolution, enabling the observation of subtle alterations in plastic morphology, which allow the study of microbial interactions with the surfaces of plastic polymers and the formation of biofilm [8]. Changes in surface morphology serve as tangible measures of the biodegradation process. This technique allows for seeing more subtle changes in the structure than observation under a classical optical microscope; however, this method is more expensive and time-consuming, and it requires a significant effort for analysis [23]. Sample preparation is crucial for obtaining high-quality SEM images. This may involve coating the sample with a conductive layer, e.g., gold particles, to enhance imaging quality [24,25].

#### 2.1.2. Transmission Electron Microscopy (TEM)

TEM is a very-high-resolution imaging technique based on the interaction of an electron beam with the sample [26]. It allows for visualization of sample morphology and verification of the size and spherical shape of particles [27,28]. In the context of MPs detection, this powerful tool is most frequently utilized in studies examining the effects of plastic particles on model systems. For instance, TEM was useful in the investigation of the toxic effects of PS nano- and micro-plastics on the marine bacterium *Halomonas alkaliphile* [25]. In another study, TEM characterization was performed to evaluate the possible effects of PP, PE, PET, and PVC MPs on microalgae [29]. Interesting results were also obtained in determining the interaction between fluorescent PET and PP MPs with *Cassiopea andromeda* jellyfish [30]. However, this technique has limited application for the direct detection of microplastics, which are characterized by their amorphous and elementary composition. This results in weak interactions with electrons and poor contrast shown by polymers in the TEM analysis. As a solution, heavy-metal staining to enhance the detection efficiency is proposed. Moreover, in TEM, there is a limit on the thickness that the studied particles should possess for a valid analysis [23,31], and it is not suitable for accurate mass-based concentration measurements. Additionally, the process of solvent evaporation during sample preparation may lead to particle aggregation on the grid, which can lead to misleading results (larger particles masking smaller ones). Nevertheless, TEM can be combined with elemental analysis to yield qualitative information about particle chemical composition [27], or it can be used as one of multiple methods to provide a comprehensive overview of the MPs under study.

#### 2.1.3. Atomic Force Microscopy

AFM is a highly sensitive microscopic technique used to examine surface topography and roughness. Similarly to SEM, this method is extensively used in biodegradation studies of synthetic polymers, as it effectively captures surface changes induced by microbial activity. On the images, increased surface roughness and various pits and cracks can be observed as the biodegradation process progresses [32,33]. In the study by Fang et al. this method was used to indicate the degradation and surface changes of PP and PE MPs caused by bacteria obtained from mangroves. In this study, findings obtained by AFM align with results from complementary methods like SEM and FTIR, emphasizing AFM’s role in confirming degradation [34]. This technique offers several advantages, including high resolution and minimal sample preparation. Unlike electron microscopy methods (SEM or TEM), AFM does not require coating or elaborate preparation. When it comes to time efficiency, use of this method can be relatively quick for small areas of the sample; however, for large-scale surface imaging, due to the scanning process, which acquires data point by point, this method can be time-consuming [35,36].

#### 2.1.4. Fluorescence Microscopy/Nile Red (NR) Staining

Fluorescent dyes, for example, NR, are used in the staining of MPs, helping to distinguish between plastic waste and non-plastic particles or fragments in environmental samples [23,37]. NR is a dye that selectively binds to hydrophobic regions [38,39,40]. MPs detection using NR is described as cost- and time-effective and can be semi-automated for high-throughput sample analysis. Additionally, it uses readily available equipment [39,40,41]. Degradation of plastic affects the fluorescence after NR staining [41]. As biodegradation progresses, more hydrophilic by-products are produced, due to microbial activity. As the plastic becomes more susceptible to microbial degradation, the number of hydrophobic regions to which NR binds may decrease, leading to a decrease in the number of stained regions. Therefore, monitoring changes in Nile red fluorescence over time can serve as an indicator of the biodegradation process and the changing hydrophobicity/hydrophilicity of the plastic surface. Additionally, the plastic fragments can break down into smaller fragments during the biodegradation process, and the resulting MPs will be visible after NR staining [38].

### 2.2. Spectroscopic Methods

#### 2.2.1. Raman Spectroscopy

In the process of the biodegradation of plastics, the issue of polymer residues is important. Raman spectroscopy turns out to be ground-breaking and extremely helpful because it uses optical effects that directly reflect the structure and chemical conditions of molecules in living cells or tissues [42]. This analytical method uses the interaction of light with chemical bonds. It provides information on chemical structure, crystallinity, and molecular interactions. Raman spectroscopy is used in plastic degradation studies to analyze the Raman spectra of MPs and identify different types of polymers [43]. Together with FTIR, it is one of the preferred methods used to identify plastics, with a significant advantage over FTIR in the analysis of MPs particles less than 20 µm in size [43,44,45]. Raman imaging technology enables extremely efficient analysis of samples, including polymer biodegradation products. It uses a single-wavelength light source that induces Raman scattering, resulting in the acquisition of the molecular vibrations of the sample in the form of a Raman spectrum. Raman spectroscopy offers a number of benefits, including high spatial resolution, a wide spectral range, high sensitivity to non-polar functional groups, and low water interference [44,46]. The main issue in the use of Raman spectroscopy is distorted spectra caused by the fluorescence of plastic additives, which lead to a low signal-to-noise ratio in Raman spectra. Another limitation of the method’s use is in environmental samples subjected to natural weathering processes. This fact is associated with changes in the structure of the plastic surface, most often caused by oxidation processes of functional groups. Therefore, it is not recommended to use this method to analyze unknown environmental samples for identification purposes [47,48].

#### 2.2.2. X-Ray Photoelectron Spectroscopy (XPS)

XPS is a sensitive analytical technique that determines the elemental surface composition and binding states of elements by analyzing the emitted electrons [49]. XPS represents an innovative approach in methods dedicated to MPs’ detection. Allowing for quantification of elemental ratios (e.g., C/O, F/C), XPS provides insights into the presence of chemical functional groups on the surfaces of MPs and helps in identifying the polymer composition of MPs. XPS is often used in conjunction with other analytical techniques, such as FTIR and microscopy, to obtain a more comprehensive understanding of MPs’ properties. Among other applications, XPS has been used to determine the chemical composition of MPs from tea bags [50]. It was also applied in the analysis of heavy metals and other associated contaminants coexisting with MPs [51]. XPS was also used in determining the biodegradation effects of linear low-density polyethylene (LLDPE), high-density polyethylene (HDPE), and PVC. The spectra showed significant changes in oxygen content for LLDPE and HDPE and a decrease in the concentration of chloride ions combined with the weakening of C–Cl bonds in the case of PVC [25]. XPS also indicated changes during the aging of PP and HDPE films using a UV-ozone chamber. After aging, the initial oxygen content in the HDPE was lower than in the PP, making the change in surface oxygen content greater for these MPs [52]. While XPS is a powerful surface analysis technique, it has some limitations. It only reveals information about the outermost layers, limiting insights into the bulk composition of MPs. Reliable results require meticulous sample preparation due to the need to remove low-molecular-weight material, solvents, salts, and buffers. Analyzing polymers with XPS may generate weak signals for small nanoplastics, leading to potential false positives or negatives [53].

#### 2.2.3. X-Ray Diffraction (XRD)

XRD is employed for the primary characterization of material properties such as crystal structure, crystallite size, and strain. The generated diffraction patterns determined by the position, arrangement, and size of the constituents of the polymer are like fingerprints that can be compared to library data for identification [54]. Apart from understanding the crystalline nature of MPs and their identification, XRD helps to contribute to the comprehensive study of these materials in various environmental contexts. For instance, changes in crystallinity patterns can provide insights into the effects of environmental factors on the structural integrity of MPs or may indicate how MPs interact with minerals, sediments, or other substances in aquatic or soil ecosystems [55,56,57]. The XRD analysis may offer valuable insights into the alterations in the structural composition of the polymers throughout the process of biodegradation. However, changes in polymer crystallinity do not always correspond to the rate of biodegradation. For instance, in the study by Sun et al. [25], the biodegradation rates expressed as weight loss for HDPE and LLDPE were similar, but the decreases in the crystallinity of the materials were quite different (72.00% to 5.78% and 54.56% to 50.25%, respectively). Interestingly, the same study showed no significant change in the crystallinity of PVC despite the apparent degradation of the polymer (12.1% ± 2.0%). This can be explained by the unique arrangement of PVC, where chlorine atoms disrupt the symmetry. As a result, regularly aligning the chains is challenging, leading to inadequate crystallization. Crystals are formed only in the co-regular segment of PVC chains, contributing to their stable crystallinity during degradation when compared to HDPE. Thus, XRD may be useful for validating biodegradability, but it should not be relied upon exclusively.

#### 2.2.4. Fourier Transform Infrared Spectroscopy

FTIR, is a technique that uses infrared radiation for investigating the chemical structure of samples. The spectrum generated by the absorption of a wave by a molecule is obtained using the Fourier transform function [58]. The method provides details of the molecular level of the tested sample, providing valuable information in degradation studies based on changes in polymer structure for detecting the degradation and ageing of specific plastics [59]. During these degradation processes, oxidative reactions occur, resulting in the formation of new bonds (C–O, C=O, and O–H), which provide evidence of degradation [60]. However, the authors note the need for extreme caution when using FTIR to detect the level of plastic degradation using biological recycling, applying microorganisms. For such degradation methods, spectrum records can be falsified by biomass, affecting the presence of C–O, C=O, CONH, and O–H bonds [60]. So far, FTIR has been used to detect degradation in PE, PS, PP, PET, PVC, and polyurethane (PUR) [61,62,63]. The main advantages of this method are as follows: high detection sensitivity, which enables the investigation of trace amounts of material [60]; universality; the possibility of testing a wide range of samples from various origins in many forms (gas, liquid, solid) [64]; the fast analysis time (from a few seconds to a few minutes) [65]; and the possibility of quantitative analysis [66]. The limitations of the method are, in particular, the fact that the results are averaged over entire samples, without the ability to specify individual molecules, which can hinder the detailed analysis of environmental samples, difficulty in identifying mixtures present in samples, and spectral interference due to impurities present in samples. A summary of all the possible changes in the structures of particular polymers is presented in Table S1B, provided in the Supplementary Materials.

#### 2.2.5. Nanoparticle Tracking Analysis (NTA)

NTA determines the particle size distribution in samples based on the Brownian motion of individual particles. This technique combines laser light scattering microscopy with a charge-coupled device (CCD) camera, which enables the visualization of nanoparticles [27,67]. NTA exhibits the capacity to analyze particles in the size range of 30–2000 nm [68] and shows favorable results for accuracy, repeatability, and reproducibility at high and low particle number concentrations. Moreover, dust and microorganisms are easily detected in this method, and large particles have little influence on the results [67]. However, some difficulties may arise with polydisperse mixtures, resulting in underestimation of the smaller particle sizes [69]. NTA requires several parameter adjustments [67]. For instance, NTA was used to demonstrate the formation of nanoparticles during the degradation of a PS disposable coffee cup lid, monitoring the increase in particle concentration over time [68].

#### 2.2.6. Dynamic Light Scattering (DLS)

DLS is the preferred technique employed in the routine determination of nanoparticle dimensions. Similarly to NTA, the particle size is established from fluctuations in scattered light intensity due to Brownian movement [67]. Despite the low resolution achieved, it is extensively used for the measurement of particles at the nanometer-to-micrometer interface (800 nm–5 µm) [27]. When using DLS, it should be remembered that this method cannot be considered suitable for the analysis of polydisperse or non-spherical particles. DLS is characterized by a low ability to distinguish MPs from other types of particles [26]. Furthermore, DLS strongly overestimates large particles due to the dependence of light scattering intensity on particle size. However, DLS in batch mode, e.g., coupled with asymmetric flow field flow fractionation (AF4), significantly increases the resolution power of measurements [27].

### 2.3. Chemical and Analytical Methods

#### 2.3.1. Weight Loss Measurement

The most basic technique used to confirm microbial activity on plastic films is weight loss measurement. This method is widely used in assessing the effectiveness of degradation processes [24,25,70,71,72]. The approach involves measuring the reduction in weight of a plastic sample over a specified period, providing a quantitative indicator of the rate at which the material has biodegraded. The process typically begins by exposing the plastic sample to microorganisms or environmental conditions that promote biodegradation. As the microorganisms break down the polymer chains or the plastic undergoes chemical changes, the mass of the material decreases. This reduction in mass serves as a tangible measure of the effectiveness of the biodegradation process. Mass loss is presented as a percentage change in weight. The formula for calculating mass loss is presented below:

$$\%\;\mathrm{Mass}\;\mathrm{loss}=\left(\frac{W_{0}-W}{W_{0}}\right)\;\times \;100\%$$

where W_0_ is the initial weight (g) of the plastic fragment and W is the weight (g) of the plastic fragment after biodegradation

This method enables repeated weighing of the sample, so that the rate of biodegradation can be determined and compared between different microorganisms and studies [8,73,74,75].

This method is quick and inexpensive, and it does not require complex equipment or a highly experienced operator. The degree of degradation during the test can be determined using an analytical balance with high resolution (e.g., 0.0001 g) to determine the weight of the plastic fragment before and after degradation [75,76]. However, the method is not without disadvantages. For instance, it is prone to human error and may result in loss of part of the sample, especially when dealing with very small particles [77]. This may be why, in some studies, despite maintaining the same conditions, the results varied by up to a few percentage points [25,63]. Compared to other methods discussed below, it is less reliable. Nonetheless, it is a useful tool for screening organisms capable of plastic biodegradation due to its being cost-effective and labor-efficient. This method also allows one to rapidly compare plastic biodegradation efficiency for different microorganisms and select the most promising among them for further studies. However, it cannot be used as the sole determinant of the biodegradation process.

#### 2.3.2. Clear Zone Formation

The efficiency of the biodegradation of plastics by microorganisms makes it possible to observe clean zones during cultivation on an appropriate agar medium. The medium is composed of micro and macro elements, with a limited organic carbon source. This observation confirms the ability of microbes to hydrolyze the emulsified polymers, and this technique enables high-throughput screening of environmental samples [72,78]. One technique for detecting the ability of microorganisms to hydrolyze PET involves using two layers of agar medium, with the lower one containing nutrients and the upper one PET. The nutrient agar is composed of 50% R2A agar. Buffering the medium or adding pyruvate is helpful. The top layer is created by adding PET dissolved in dimethyl sulfoxide (DMSO) to ultrapure noble agar. Ultrasonic homogenization is used to obtain a uniform solution. Ultimately, the layer is slightly cloudy and allows observation of the emerging clear zones [79].

#### 2.3.3. Contact Angle Measurement (CA)

CA measurement is a simple and minimally complex technique that assesses surface wettability, influenced by cohesive and adhesive energies [80]. This measurement involves applying a liquid droplet to a solid surface at the equilibrium point among the solid, liquid, and gaseous phases. The CA is influenced by the chemical properties of the solid surface, including polar and dispersed interactions, as well as its physical properties, such as roughness. Small contact angles (<90°) suggest high wettability, indicating that liquids spread easily on the surface [81]. Overall, CA measurement is a valuable tool in MPs research, contributing to the broader understanding of the environmental fate, interactions, and surface characteristics of these particles. CA is frequently employed to evaluate the effects of the aging and weathering of MPs in various environmental conditions. Increased hydrophilicity has also been observed in the case of PE sheets, as well as HDPE and LLDPE films after incubation with microorganisms [25,74]. Furthermore, the wettability of MPs can influence the behavior of aquatic and soil environments. It was observed that the soil CA was increased when the MPs concentration reached 2% of dry soil weight [82]. Moreover, highly wettable surfaces may promote interactions with water and other substances, affecting the fate and transport of MPs. CA measurements can also be instrumental in studying the effects of surface modifications on MPs [80]. Generally, plastics such as PE, PP, and PVC are characterized by strong hydrophobicity. By introducing hydrophilic functional groups to the plastic structure, the hydrophobic nature can be decreased. Greater hydrophilicity makes the material more susceptible to degradation [75]. In combination with other techniques, CA measurements may contribute to the identification of specific polymers associated with MPs [80].

#### 2.3.4. Thermogravimetric Analysis (TGA)

TGA is a thermal analysis technique that determines the change in mass of a sample as a function of temperature or time. TGA signals provide a profile of the thermal degradation patterns; thus, they can be easily used to identify the type of polymer present in MPs samples [83]. Identification and/or quantification of polymers through TGA requires minimal or no sample pre-treatment to separate MPs from their environmental matrix. Due to the rapidity and facility in providing chemical identification of polymers, various TGA-based methods for the analysis of MPs are being evaluated, such as a combination of TGA and DSC [84] or FTIR [85]. A fairly significant limitation of TGA is the possibility of overlapping phase transition signals, such as those of polyurethane (PU) and PET, which results in difficulties in distinguishing them [84].

#### 2.3.5. Differential Scanning Calorimetry (DSC)

DSC is a thermal analysis technique that measures the heat flow associated with physical and chemical changes in a sample as a function of temperature [86]. DSC can identify changes in the structures of polymers and provides information on various thermal properties, including the glass transition temperature, melting point, crystallization temperature, and heat capacity [83,87]. The quantification and identification are performed via the DSC signal using the area of the melting peak and its relation to the sample mass [84]. While DSC is not a direct detection method for MPs, it plays a crucial role in characterizing the thermal properties of the polymers associated with MPs particles. It helps to characterize and quantify the polymer mixtures, offering insights into the thermal behavior of individual components, where each polymer’s distinctive melting point (Tm) is identified. This approach is valuable in the identification of MPs fragments in environmental samples that cannot be detected by the naked eye and are difficult to identify with FT-IR and Raman spectroscopy [88]. Generally, DSC is effective for analyzing polymers with crystalline components, such as PE, PP, PA, and PET, but it does not apply to amorphous polymers, such as PS [89].

#### 2.3.6. Pyrolysis-Gas Chromatography/Mass Spectrometry

One of the methods developed for the identification and quantification of polymers is Pyr-GC/MS. Although it has never been claimed to provide proof of plastic biodegradation, it does have such potential. By detecting the MPs in the samples, it should be possible to validate the efficiency of the biodegradation process, by measuring the samples at the beginning and the end of the biodegradation process in a liquid culture of microorganisms. Originally, this method was used to validate environmental samples for the quantification and qualification of MPs pollutants. It has been used in the detection of MPs in live organisms [90], human blood [91], water [92], soil [93], and air [94]. This method allows for the detection of many polymers such as PE, PP, PS, PU, PET, and PVC, allowing for the detection of MPs in the range of 0.1 (PU) to 9.1 μg (PE) [92]. Before the Py-GC/MS analysis, the samples must undergo digestion (hydrogen peroxide solution (H_2_O_2_), 10% potassium hydroxide (KOH), and others) to remove organic matter. Most of the described methods rely on coupled pyrolysis using a Frontier-Lab pyrolyzer (Koriyama, Japan) with gas chromatography–mass spectrometers from different brands, such as Shimadzu (Kyoto, Japan), Agilent Technologies (Santa Clara, CA, USA), etc. The optimized Pyr-GC/MS method requires a pyrolysis temperature of 600 [92] to 700 °C [95], with the Pyr-GC transfer line set at 300 °C. The advantages of this method are high measurement sensitivity, enabling the identification of copolymers such as PE-PP, and a very small particle size range. On the other hand, this method requires costly equipment, experienced staff, and the time-consuming preparation of samples. In summary, this method still requires optimization as a tool for polymer biodegradation, but it has potential for application in this type of study.

#### 2.3.7. High-Performance Liquid Chromatography (HPLC)/Ultra-Performance Liquid Chromatography (UPLC)

UPL and HPLC are among the most common methods for the detection of plastic degradation efficiency. The main difference between HPLC and UPLC is the column filling particle size. UPLC uses particles ≤ 2 µm, while HPLC uses particles of 3 µm to 5 µm. UPLC analysis enables reductions in analysis time, improved detection sensitivity, increased resolution due to reduced dead volume, and the adaptability to operate at pressures higher than in a standard HPLC system. An HPLC system can operate at a maximum pressure of 6000 psi, while with UPLC, the achievable values reach 15,000 psi [96]. To date, U-HPLC methods have been applied to degradation products of poly(ethylene terephthalate) (PET) and polystyrene (PS). The estimation of the degradation capacity for PET is evaluated via the quantitative and qualitative analysis of samples, taking into the consideration compounds such as bis(2-hydroxyethyl) terephthalate (BHET), mono(2-hydroxyethyl) terephthalic acid (MHET), terephthalic acid (TPA), and ethylene glycol (EG) [21,97]. During PS degradation, many different compounds are formed. Atiq et al. focused on 2-phenylethanol, 1-phenyl-1, 2-ethanediol, phenylacetaldehyde, styrene oxide, and styrene, which can be determined via HPLC [98]. Styrene can be analyzed using the Zorbax ODS, which is a silica-based, C18-type column, as well as 2-phenylethanol (with the ZORBAX Eclipse Plus C18 column, Agilent Technologies, Santa Clara, CA, USA) [99] and phenylacetaldehyde (with the BEH C18 or Discovery C18 column) [100]. A detailed description of the detection conditions and methods is presented in Table S1A, provided in the Supplementary Materials.

#### 2.3.8. A Multi-Aspect Comparison of MPs Detection Methods

**Figure 4:**
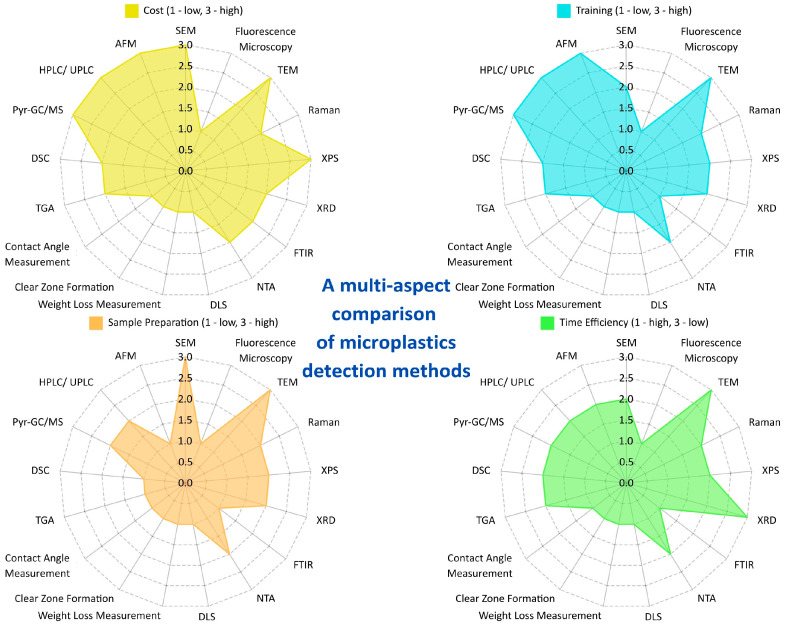
A multi-aspect comparison of MPs detection methods [graph created with the free version of canva.com].

As stated in this review, there is still no single sufficiently reliable method to assess the biodegradation of plastics, but some methods offer significantly more advantages than others. The advantages and disadvantages of each method are discussed in detail in Table S1A–C, in the Supplementary Materials in this review. In addition, in the figure below, the methods are compared in terms of four important factors affecting their suitability for plastic degradation studies. A comparison of the methods listed in this review in terms of cost, the need for a highly experienced operator, sample preparation, and time efficiency are summarized in Figure 4. The methods presented vary significantly in terms of these criteria: high-end techniques such as TEM, SEM, and AFM provide detailed insights into the surfaces of polymer fragments but are associated with high costs and extensive training requirements, making them ideal for advanced confirmatory analyses. On the other hand, low-cost methods such as mass-loss measurement, contact angle, and fluorescence microscopy are more accessible and efficient, but often lack specificity and can be prone to operator bias. Spectroscopic approaches, including FTIR and Raman spectroscopy, offer a practical balance, providing reliable polymer identification with moderate effort and resources, but cannot be used as the only sufficient method to determine plastic degradation. For complex biodegradation studies, it is recommended to combine simple screening techniques (e.g., clear zone formation or contact angle) with confirmatory analytical methods, such as FTIR or Raman, to ensure the reliability of the obtained results.

## 3. Conclusions

Here, we have summarized the most common methods used in the study of MPs detection and plastic biodegradation. Researchers have to decide which technique is appropriate for each step in the study, from basic, cheap, and uncomplicated methods such as clear zone formation or weight loss measurement, which allow for the rapid identification of microorganisms with potential for polymer biodegradation, to more complicated, but more reliable methods confirming changes in the surfaces of films or the quantity of MPs in samples. However, it should be taken into account that there is still no single sufficiently reliable method for rapid and error-free identification of plastic degradation. It is necessary to improve existing methods and develop new ones that will be reliable and practical tools in identifying plastic biodegradation. For the reliable identification of plastic biodegradation, a combination of several methods for measuring weight loss with microscopic and analytical methods is necessary.

## Data Availability

Dataset available on request from the authors.

## Supplementary Materials

The following supporting information can be downloaded at https://www.mdpi.com/article/10.3390/molecules30112462/s1: Table S1A: Comparison of methods for identification and characterization of microplastics with examples of applications and advantages and limitations of these methods; Table S1B. Comparison of methods for identification and characterization of microplastics with examples of applications and advantages and limitations of these 12 methods; Table S1C. Comparison of methods for identification and characterization of microplastics with examples of applications and advantages and limitations of these 16 methods. References [101,102,103,104,105,106,107,108,109,110,111,112,113,114,115,116,117,118,119,120,121] are cited in the Supplementary Materials.

## Abbreviations

The following abbreviations are used in this manuscript:

MPs	microplastics
PET	polyethylene terephthalate
PE	polyethylene
LLDPE	linear low-density polyethylene
HDPE	high-density polyethylene
PP	polypropylene
PUR	polyurethane
PS	polystyrene
PVC	polyvinyl chloride
PLA	polylactic acid
PBS	polybutylene succinate
PHA	polyhydroxyalkanoates
PCL	polycaprolactone
SEM	scanning electron microscopy
AFM	atomic force microscopy
TEM	transmission electron microscopy
HPLC	high-performance liquid chromatography
UPLC	ultra-performance liquid chromatography
FTIR	Fourier transform infrared spectroscopy
Pyr-GC/MS	pyrolysis–gas chromatography–mass spectrometry
XPS	X-ray photoelectron spectroscopy
XRD	X-ray diffraction
DSC	differential scanning calorimetry
CA	contact angle
TGA	thermogravimetric analysis
NTA	nanoparticle tracking analysis
DLS	dynamic light scattering
NR	Nile red

## References

[1] Kim M.S., Chang H., Zheng L., Yan Q., Pfleger B.F., Klier J., Nelson K., Majumder E.L., Huber G.W. (2023). A Review of Biodegradable Plastics: Chemistry, Applications, Properties, and Future Research Needs. Chem. Rev..

[2] PlasticEurope (2023). Plastic-the Facts 2023. An Analysis of European Plastic Production, Demand and Waste Data. https://plasticseurope.org/knowledge-hub/plastics-the-fast-facts-2023/.

[3] Kaur P., Singh K., Singh B. (2022). Microplastics in soil: Impacts and microbial diversity and degradation. Pedosphere.

[4] Urbanek A.K., Rymowicz W., Mironczuk A.M. (2018). Degradation of plastics and plastic-degrading bacteria in cold marine habitats. Appl. Microbiol. Biotechnol..

[5] Thompson R.C., Olsen Y., Mitchell R.P., Davis A., Rowland S.J., John A.W., McGonigle D., Russell A.E. (2004). Lost at sea: Where is all the plastic?. Science.

[6] Gigault J., Halle A.T., Baudrimont M., Pascal P.Y., Gauffre F., Phi T.L., El Hadri H., Grassl B., Reynaud S. (2018). Current opinion: What is a nanoplastic?. Environ. Pollut..

[7] Urbanek A.K., Kosiorowska K.E., Mironczuk A.M. (2021). Current Knowledge on Polyethylene Terephthalate Degradation by Genetically Modified Microorganisms. Front. Bioeng. Biotechnol..

[8] Yoshida S., Hiraga K., Takehana T., Taniguchi I., Yamaji H., Maeda Y., Toyohara K., Miyamoto K., Kimura Y., Oda K. (2016). A bacterium that degrades and assimilates poly (ethylene terephthalate). Science.

[9] Wilkes R.A., Aristilde L. (2017). Degradation and metabolism of synthetic plastics and associated products by *Pseudomonas* sp.: Capabilities and challenges. J. Appl. Microbiol..

[10] Zhu J., Dong G., Feng F., Ye J., Liao C.H., Wu C.H., Chen S.C. (2023). Microplastics in the soil environment: Focusing on the sources, its transformation and change in morphology. Sci. Total Environ..

[11] He W., Liu S., Zhang W., Yi K., Zhang C., Pang H., Huang D., Huang J., Li X. (2023). Recent advances on microplastic aging: Identification, mechanism, influence factors, and additives release. Sci. Total Environ..

[12] Lei K., Qiao F., Liu Q., Wei Z., Qi H., Cui S., Yue X., Deng Y., An L. (2017). Microplastics releasing from personal care and cosmetic products in China. Mar. Pollut. Bull..

[13] Wagner M., Scherer C., Alvarez-Munoz D., Brennholt N., Bourrain X., Buchinger S., Fries E., Grosbois C., Klasmeier J., Marti T. (2014). Microplastics in freshwater ecosystems: What we know and what we need to know. Environ. Sci. Eur..

[14] Sekudewicz I., Dabrowska A.M., Syczewski M.D. (2021). Microplastic pollution in surface water and sediments in the urban section of the Vistula River (Poland). Sci. Total Environ..

[15] Wang Y., Zhao J., Fu Z., Guan D., Zhang D., Zhang H., Zhang Q., Xie J., Sun Y., Wang D. (2024). Innovative overview of the occurrence, aging characteristics, and ecological toxicity of microplastics in environmental media. Environ. Pollut..

[16] Schwabl P., Koppel S., Konigshofer P., Bucsics T., Trauner M., Reiberger T., Liebmann B. (2019). Detection of Various Microplastics in Human Stool: A Prospective Case Series. Ann. Intern. Med..

[17] He D., Zhang Y., Gao W. (2021). Micro(nano)plastic contaminations from soils to plants: Human food risks. Curr. Opin. Food Sci..

[18] Limonta G., Mancia A., Benkhalqui A., Bertolucci C., Abelli L., Fossi M.C., Panti C. (2019). Microplastics induce transcriptional changes, immune response and behavioral alterations in adult zebrafish. Sci. Rep..

[19] Mak C.W., Ching-Fong Yeung K., Chan K.M. (2019). Acute toxic effects of polyethylene microplastic on adult zebrafish. Ecotoxicol. Environ. Saf..

[20] Yang J., Yang Y., Wu W.M., Zhao J., Jiang L. (2014). Evidence of polyethylene biodegradation by bacterial strains from the guts of plastic-eating waxworms. Environ. Sci. Technol..

[21] Kosiorowska K.E., Biniarz P., Dobrowolski A., Leluk K., Mironczuk A.M. (2022). Metabolic engineering of Yarrowia lipolytica for poly(ethylene terephthalate) degradation. Sci. Total Environ..

[22] Page M.J., McKenzie J.E., Bossuyt P.M., Boutron I., Hoffmann T.C., Mulrow C.D., Shamseer L., Tetzlaff J.M., Akl E.A., Brennan S.E. (2021). The PRISMA 2020 statement: An updated guideline for reporting systematic reviews. BMJ.

[23] Mariano S., Tacconi S., Fidaleo M., Rossi M., Dini L. (2021). Micro and Nanoplastics Identification: Classic Methods and Innovative Detection Techniques. Front. Toxicol..

[24] Auta H.S., Emenike C.U., Jayanthi B., Fauziah S.H. (2018). Growth kinetics and biodeterioration of polypropylene microplastics by Bacillus sp. and *Rhodococcus* sp. isolated from mangrove sediment. Mar. Pollut. Bull..

[25] Sun W., Zhang Y., Zhang H., Wu H., Liu Q., Yang F., Hou M., Qi Y., Zhang W. (2024). Exploitation of Enterobacter hormaechei for biodegradation of multiple plastics. Sci. Total Environ..

[26] Huber M.J., Ivleva N.P., Booth A.M., Beer I., Bianchi I., Drexel R., Geiss O., Mehn D., Meier F., Molska A. (2023). Physicochemical characterization and quantification of nanoplastics: Applicability, limitations and complementarity of batch and fractionation methods. Anal. Bioanal. Chem..

[27] Caputo F., Vogel R., Savage J., Vella G., Law A., Della Camera G., Hannon G., Peacock B., Mehn D., Ponti J. (2021). Measuring particle size distribution and mass concentration of nanoplastics and microplastics: Addressing some analytical challenges in the sub-micron size range. J. Colloid Interface Sci..

[28] Correia M., Loeschner K. (2018). Detection of nanoplastics in food by asymmetric flow field-flow fractionation coupled to multi-angle light scattering: Possibilities, challenges and analytical limitations. Anal. Bioanal. Chem..

[29] Song C., Liu Z., Wang C., Li S., Kitamura Y. (2020). Different interaction performance between microplastics and microalgae: The bio-elimination potential of Chlorella sp. L38 and Phaeodactylum tricornutum MASCC-0025. Sci. Total Environ..

[30] Caldwell J., Loussert-Fonta C., Toullec G., Heidelberg Lyndby N., Haenni B., Taladriz-Blanco P., Espina B., Rothen-Rutishauser B., Petri-Fink A. (2023). Correlative Light, Electron Microscopy and Raman Spectroscopy Workflow To Detect and Observe Microplastic Interactions with Whole Jellyfish. Environ. Sci. Technol..

[31] Kalaronis D., Ainali N.M., Evgenidou E., Kyzas G.Z., Yang X., Bikiaris D.N., Lambropoulou D.A. (2022). Microscopic techniques as means for the determination of microplastics and nanoplastics in the aquatic environment: A concise review. Green Anal. Chem..

[32] Woo S., Song I., Cha Hyung J. (2020). Fast and Facile Biodegradation of Polystyrene by the Gut Microbial Flora of Plesiophthalmus davidis Larvae. Appl. Environ. Microbiol..

[33] Roy R., Mukherjee G., Das Gupta A., Tribedi P., Sil A.K. (2021). Isolation of a soil bacterium for remediation of polyurethane and low-density polyethylene: A promising tool towards sustainable cleanup of the environment. 3 Biotech.

[34] Fang X., Cai Z., Wang X., Liu Z., Lin Y., Li M., Gong H., Yan M. (2024). Isolation and Identification of Four Strains of Bacteria with Potential to Biodegrade Polyethylene and Polypropylene from Mangrove. Microorganisms.

[35] Li W., Luo Y., Pan X., He D., Luo Y. (2020). Identification and Characterization Methods for Microplastics Basing on Spatial Imaging in Micro-/Nanoscales. Microplastics in Terrestrial Environments: Emerging Contaminants and Major Challenges.

[36] Obrador-Viel T., Zadjelovic V., Nogales B., Bosch R., Christie-Oleza J.A. (2024). Assessing microbial plastic degradation requires robust methods. Microb. Biotechnol..

[37] Shruti V.C., Perez-Guevara F., Roy P.D., Kutralam-Muniasamy G. (2022). Analyzing microplastics with Nile Red: Emerging trends, challenges, and prospects. J. Hazard. Mater..

[38] Bianco A., Carena L., Peitsaro N., Sordello F., Vione D., Passananti M. (2023). Rapid detection of nanoplastics and small microplastics by Nile-Red staining and flow cytometry. Environ. Chem. Lett..

[39] Erni-Cassola G., Gibson M.I., Thompson R.C., Christie-Oleza J.A. (2017). Lost, but Found with Nile Red: A Novel Method for Detecting and Quantifying Small Microplastics (1 mm to 20 μm) in Environmental Samples. Environ. Sci. Technol..

[40] Shim W.J., Song Y.K., Hong S.H., Jang M. (2016). Identification and quantification of microplastics using Nile Red staining. Mar. Pollut. Bull..

[41] Meyers N., Catarino A.I., Declercq A.M., Brenan A., Devriese L., Vandegehuchte M., De Witte B., Janssen C., Everaert G. (2022). Microplastic detection and identification by Nile red staining: Towards a semi-automated, cost- and time-effective technique. Sci. Total Environ..

[42] Elert A.M., Becker R., Duemichen E., Eisentraut P., Falkenhagen J., Sturm H., Braun U. (2017). Comparison of different methods for MP detection: What can we learn from them, and why asking the right question before measurements matters?. Environ. Pollut..

[43] Ramanna S., Morozovskii D., Swanson S., Bruneau J. (2023). Machine Learning of Polymer Types From the Spectral Signature of Raman Spectroscopy Microplastics Data. Adv. Artif. Intell. Mach. Learn..

[44] Araujo C.F., Nolasco M.M., Ribeiro A.M.P., Ribeiro-Claro P.J.A. (2018). Identification of microplastics using Raman spectroscopy: Latest developments and future prospects. Water Res..

[45] Cabernard L., Roscher L., Lorenz C., Gerdts G., Primpke S. (2018). Comparison of Raman and Fourier Transform Infrared Spectroscopy for the Quantification of Microplastics in the Aquatic Environment. Environ. Sci. Technol..

[46] Anger P.M., von der Esch E., Baumann T., Elsner M., Niessner R., Ivleva N.P. (2018). Raman microspectroscopy as a tool for microplastic particle analysis. TrAC Trends Anal. Chem..

[47] Cai L., Wang J., Peng J., Wu Z., Tan X. (2018). Observation of the degradation of three types of plastic pellets exposed to UV irradiation in three different environments. Sci. Total Environ..

[48] Nava V., Frezzotti M.L., Leoni B. (2021). Raman Spectroscopy for the Analysis of Microplastics in Aquatic Systems. Appl. Spectrosc..

[49] Mather R.R., Wei Q. (2009). 13—Surface modification of textiles by plasma treatments. Surface Modification of Textiles.

[50] Hernandez L.M., Xu E.G., Larsson H.C.E., Tahara R., Maisuria V.B., Tufenkji N. (2019). Plastic Teabags Release Billions of Microparticles and Nanoparticles into Tea. Environ. Sci. Technol..

[51] Melo-Agustin P., Kozak E.R., de Jesus Perea-Flores M., Mendoza-Perez J.A. (2022). Identification of microplastics and associated contaminants using ultra high resolution microscopic and spectroscopic techniques. Sci. Total Environ..

[52] Bhagat K., Barrios A.C., Rajwade K., Kumar A., Oswald J., Apul O., Perreault F. (2022). Aging of microplastics increases their adsorption affinity towards organic contaminants. Chemosphere.

[53] Sobhani Z., Zhang X., Gibson C., Naidu R., Megharaj M., Fang C. (2020). Identification and visualisation of microplastics/nanoplastics by Raman imaging (i): Down to 100 nm. Water Res..

[54] Moura D.S., Pestana C.J., Moffat C.F., Hui J., Irvine J.T.S., Lawton L.A. (2023). Characterisation of microplastics is key for reliable data interpretation. Chemosphere.

[55] Fu Q., Tan X., Ye S., Ma L., Gu Y., Zhang P., Chen Q., Yang Y., Tang Y. (2021). Mechanism analysis of heavy metal lead captured by natural-aged microplastics. Chemosphere.

[56] Thakur B., Singh J., Singh J., Angmo D., Vig A.P. (2023). Identification and characterization of extracted microplastics from agricultural soil near industrial area: FTIR and X-ray diffraction method. Environ. Qual. Manag..

[57] Zhang S., Han B., Sun Y., Wang F. (2020). Microplastics influence the adsorption and desorption characteristics of Cd in an agricultural soil. J. Hazard. Mater..

[58] Fabris H.J., Knauss W.G., Allen G., Bevington J.C. (1989). 5—Synthetic Polymer Adhesives. Comprehensive Polymer Science and Supplements.

[59] Sorasan C., Ortega-Ojeda F.E., Rodríguez A., Rosal R. (2022). Modelling the Photodegradation of Marine Microplastics by Means of Infrared Spectrometry and Chemometric Techniques. Microplastics.

[60] Sandt C., Waeytens J., Deniset-Besseau A., Nielsen-Leroux C., Rejasse A. (2021). Use and misuse of FTIR spectroscopy for studying the bio-oxidation of plastics. Spectrochim. Acta A Mol. Biomol. Spectrosc..

[61] Chen Y., Wang B. (2022). Effect of Diatomite on the Thermal Degradation Behavior of Polypropylene and Formation of Graphene Products. Polymers.

[62] Dimassi S.N., Hahladakis J.N., Daly Yahia M.N., Ahmad M.I., Sayadi S., Al-Ghouti M.A. (2023). Insights into the degradation mechanism of PET and PP under marine conditions using FTIR. J. Hazard. Mater..

[63] Jeyakumar D., Chirsteen J., Doble M. (2013). Synergistic effects of pretreatment and blending on fungi mediated biodegradation of polypropylenes. Bioresour. Technol..

[64] Kowalczuk D., Pitucha M. (2019). Application of FTIR Method for the Assessment of Immobilization of Active Substances in the Matrix of Biomedical Materials. Materials.

[65] Rodriguez-Saona L.E., Allendorf M.E. (2011). Use of FTIR for rapid authentication and detection of adulteration of food. Annu. Rev. Food Sci. Technol..

[66] Mayerhofer T.G., Pahlow S., Popp J. (2020). The Bouguer-Beer-Lambert Law: Shining Light on the Obscure. Chemphyschem.

[67] Filipe V., Hawe A., Jiskoot W. (2010). Critical evaluation of Nanoparticle Tracking Analysis (NTA) by NanoSight for the measurement of nanoparticles and protein aggregates. Pharm. Res..

[68] Lambert S., Wagner M. (2016). Characterisation of nanoplastics during the degradation of polystyrene. Chemosphere.

[69] Ruud J.B.P., Relou E., Sijtsma E.L.E., Undas A.K. (2024). Evaluation of Nanoparticle Tracking Analysis (NTA) for the Measurement of Nanoplastics in Drinking Water. Research Square.

[70] Arkatkar A., Arutchelvi J., Bhaduri S., Uppara P.V., Doble M. (2009). Degradation of unpretreated and thermally pretreated polypropylene by soil consortia. Int. Biodeterior. Biodegrad..

[71] Vimala P.P., Mathew L. (2016). Biodegradation of Polyethylene Using Bacillus Subtilis. Procedia Technol..

[72] Urbanek A.K., Rymowicz W., Strzelecki M.C., Kociuba W., Franczak L., Mironczuk A.M. (2017). Isolation and characterization of Arctic microorganisms decomposing bioplastics. AMB Express.

[73] Taghavi N., Udugama I.A., Zhuang W.Q., Baroutian S. (2021). Challenges in biodegradation of non-degradable thermoplastic waste: From environmental impact to operational readiness. Biotechnol. Adv..

[74] Hou L., Xi J., Liu J., Wang P., Xu T., Liu T., Qu W., Lin Y.B. (2022). Biodegradability of polyethylene mulching film by two Pseudomonas bacteria and their potential degradation mechanism. Chemosphere.

[75] Xiang P., Zhang Y., Zhang T., Wu Q., Zhao C., Li Q. (2023). A novel bacterial combination for efficient degradation of polystyrene microplastics. J. Hazard. Mater..

[76] Park S.Y., Kim C.G. (2019). Biodegradation of micro-polyethylene particles by bacterial colonization of a mixed microbial consortium isolated from a landfill site. Chemosphere.

[77] Wei R., Tiso T., Bertling J., O’Connor K., Blank L.M., Bornscheuer U.T. (2020). Possibilities and limitations of biotechnological plastic degradation and recycling. Nat. Catal..

[78] Urbanek A.K., Strzelecki M.C., Mironczuk A.M. (2021). The potential of cold-adapted microorganisms for biodegradation of bioplastics. Waste Manag..

[79] Charnock C. (2021). A simple and novel method for the production of polyethylene terephthalate containing agar plates for the growth and detection of bacteria able to hydrolyze this plastic. J. Microbiol. Methods.

[80] Chiou C.-H., Hsieh S.-J. (2015). Empirical study and prediction of contact angle and surface free energy of commonly used plastics with pillar-like structure. Surf. Interface Anal..

[81] Benke A., Sonnenberg J., Oelschlägel K., Schneider M., Lux M., Potthoff A. (2022). Wettability after Artificial and Natural Weathering of Polyethylene Terephthalate. Environments.

[82] Shafea L., Felde V.J.M.N.L., Woche S.K., Bachmann J., Peth S. (2023). Microplastics effects on wettability, pore sizes and saturated hydraulic conductivity of a loess topsoil. Geoderma.

[83] Sorolla-Rosario D., Llorca-Porcel J., Pérez-Martínez M., Lozano-Castelló D., Bueno-López A. (2022). Study of microplastics with semicrystalline and amorphous structure identification by TGA and DSC. J. Environ. Chem. Eng..

[84] Majewsky M., Bitter H., Eiche E., Horn H. (2016). Determination of microplastic polyethylene (PE) and polypropylene (PP) in environmental samples using thermal analysis (TGA-DSC). Sci. Total Environ..

[85] Yu J., Wang P., Ni F., Cizdziel J., Wu D., Zhao Q., Zhou Y. (2019). Characterization of microplastics in environment by thermal gravimetric analysis coupled with Fourier transform infrared spectroscopy. Mar. Pollut. Bull..

[86] Gill P., Moghadam T.T., Ranjbar B. (2010). Differential scanning calorimetry techniques: Applications in biology and nanoscience. J. Biomol. Tech..

[87] Rodriguez Chialanza M., Sierra I., Perez Parada A., Fornaro L. (2018). Identification and quantitation of semi-crystalline microplastics using image analysis and differential scanning calorimetry. Environ. Sci. Pollut. Res. Int..

[88] Shabaka S.H., Ghobashy M., Marey R.S. (2019). Identification of marine microplastics in Eastern Harbor, Mediterranean Coast of Egypt, using differential scanning calorimetry. Mar. Pollut. Bull..

[89] Bitter H., Lackner S. (2021). Fast and easy quantification of semi-crystalline microplastics in exemplary environmental matrices by differential scanning calorimetry (DSC). Chem. Eng. J..

[90] Dehaut A., Cassone A.L., Frere L., Hermabessiere L., Himber C., Rinnert E., Riviere G., Lambert C., Soudant P., Huvet A. (2016). Microplastics in seafood: Benchmark protocol for their extraction and characterization. Environ. Pollut..

[91] Leslie H.A., van Velzen M.J.M., Brandsma S.H., Vethaak A.D., Garcia-Vallejo J.J., Lamoree M.H. (2022). Discovery and quantification of plastic particle pollution in human blood. Environ. Int..

[92] Santos L., Insa S., Arxe M., Buttiglieri G., Rodriguez-Mozaz S., Barcelo D. (2023). Analysis of microplastics in the environment: Identification and quantification of trace levels of common types of plastic polymers using pyrolysis-GC/MS. MethodsX.

[93] Dierkes G., Lauschke T., Becher S., Schumacher H., Foldi C., Ternes T. (2019). Quantification of microplastics in environmental samples via pressurized liquid extraction and pyrolysis-gas chromatography. Anal. Bioanal. Chem..

[94] Morioka T., Tanaka S., Kohama-Inoue A., Watanabe A. (2024). The quantification of the airborne plastic particles of 0.43–11 μm: Procedure development and application to atmospheric environment. Chemosphere.

[95] Hermabessiere L., Himber C., Boricaud B., Kazour M., Amara R., Cassone A.L., Laurentie M., Paul-Pont I., Soudant P., Dehaut A. (2018). Optimization, performance, and application of a pyrolysis-GC/MS method for the identification of microplastics. Anal. Bioanal. Chem..

[96] Swetha Sri R., Bhavya S.K., Mounika C. (2020). A review on comparative study of HPLC and UPLC. Res. J. Pharm. Technol..

[97] Kosiorowska K.E., Moreno A.D., Iglesias R., Leluk K., Mironczuk A.M. (2022). Production of PETase by engineered Yarrowia lipolytica for efficient poly(ethylene terephthalate) biodegradation. Sci. Total Environ..

[98] Atiq N., Garba A., Ali M.I., Andleeb S. (2010). Isolation and identification of polystyrene biodegrading bacteria from soil Study of Pyocyanin induced pathogenicity of Pseudomonas aeruginosa. Artic. Afr. J. Microbiol. Res..

[99] Gu Y., Ma J., Zhu Y., Xu P. (2020). Refactoring Ehrlich Pathway for High-Yield 2-Phenylethanol Production in Yarrowia lipolytica. ACS Synth. Biol..

[100] Xu Y., Ye H., Dong H., Zeng X., Yang J., Xiao G., Bai W., Wu J., He Q., Xian Y. (2023). Determination of 2-amino-1-methyl-6-phenylimidazole [4, 5-b] pyridine (PhIP) and its precursors and possible intermediates in a chemical model system and roast pork. LWT.

[101] Yang S.S., Ding M.Q., He L., Zhang C.H., Li Q.X., Xing D.F., Cao G.L., Zhao L., Ding J., Ren N.Q. (2021). Biodegradation of polypropylene by yellow mealworms (*Tenebrio molitor*) and superworms (*Zophobas atratus*) via gut-microbe-dependent depolymerization. Sci. Total Environ..

[102] Ekvall M.T., Gimskog I., Hua J., Kelpsiene E., Lundqvist M., Cedervall T. (2022). Size fractionation of high-density polyethylene breakdown nanoplastics reveals different toxic response in Daphnia magna. Sci. Rep..

[103] Tomoda B.T., Yassue-Cordeiro P.H., Ernesto J.V., Lopes P.S., Péres L.O., da Silva C.F., de Moraes M.A., de Moraes M.A., da Silva C.F., Vieira R.S. (2020). Chapter 3—Characterization of biopolymer membranes and films: Physicochemical, mechanical, barrier, and biological properties. Biopolymer Membranes and Films.

[104] Sun X., Chen B., Li Q., Liu N., Xia B., Zhu L., Qu K. (2018). Toxicities of polystyrene nano- and microplastics toward marine bacterium *Halomonas alkaliphila*. Sci. Total Environ..

[105] Moraz A., Breider F. (2021). Detection and Quantification of Nonlabeled Polystyrene Nanoparticles Using a Fluorescent Molecular Rotor. Anal. Chem..

[106] Maddison C., Sathish C.I., Lakshmi D., Wayne O., Palanisami T. (2023). An advanced analytical approach to assess the long-term degradation of microplastics in the marine environment. Npj Mater. Degrad..

[107] Wu Q., Tao H., Wong M.H. (2019). Feeding and metabolism effects of three common microplastics on *Tenebrio molitor* L.. Environ. Geochem. Health.

[108] Singh R.K., Ruj B., Sadhukhan A., Gupta P. (2020). A TG-FTIR investigation on the co-pyrolysis of the waste HDPE, PP, PS and PET under high heating conditions. J. Energy Inst..

[109] Alzuhairi M. (2016). Chemical Recycling of Polyethylene Terephthalate (PET) as Additive for Asphalt. ZANCO J. Pure Appl. Sci..

[110] Afonso E., Martínez-Gómez A., Huerta A., Tiemblo P., García N. (2022). Facile Preparation of Hydrophobic PET Surfaces by Solvent Induced Crystallization. Coatings.

[111] Ojha N., Pradhan N., Singh S., Barla A., Shrivastava A., Khatua P., Rai V., Bose S. (2017). Evaluation of HDPE and LDPE degradation by fungus, implemented by statistical optimization. Sci. Rep..

[112] Jiao L., Xiao H., Wang Q., Sun J. (2013). Thermal degradation characteristics of rigid polyurethane foam and the volatile products analysis with TG-FTIR-MS. Polym. Degrad. Stab..

[113] Gamerith C., Acero E.H., Pellis A., Ortner A., Vielnascher R., Luschnig D., Zartl B., Haernvall K., Zitzenbacher S., Strohmeier G. (2016). Improving enzymatic polyurethane hydrolysis by tuning enzyme sorption. Polym. Degrad. Stab..

[114] Yaseen A.A., Yousif E., Al-Tikrity E.T.B., El-Hiti G.A., Kariuki B.M., Ahmed D.S., Bufaroosha M. (2021). FTIR, Weight, and Surface Morphology of Poly(vinyl chloride) Doped with Tin Complexes Containing Aromatic and Heterocyclic Moieties. Polymers.

[115] Wang Z., Wei R., Wang X., He J., Wang J. (2018). Pyrolysis and Combustion of Polyvinyl Chloride (PVC) Sheath for New and Aged Cables via Thermogravimetric Analysis-Fourier Transform Infrared (TG-FTIR) and Calorimeter. Materials.

[116] Karkanorachaki K., Tsiota P., Dasenakis G., Syranidou E., Kalogerakis N. (2022). Nanoplastic Generation from Secondary PE Microplastics: Microorganism-Induced Fragmentation. Microplastics.

[117] Kosiorowska K., Mironczuk A. (2022). Enhanced poly (ethylene terephthalate) degradation by modified Yarrowia lipolytica strains. FEBS OPEN Bio.

[118] Hu X., Thumarat U., Zhang X., Tang M., Kawai F. (2010). Diversity of polyester-degrading bacteria in compost and molecular analysis of a thermoactive esterase from Thermobifida alba AHK119. Appl. Microbiol. Biotechnol..

[119] Tiso T., Narancic T., Wei R., Pollet E., Beagan N., Schröder K., Honak A., Jiang M., Kenny S.T., Wierckx N. (2021). Towards bio-upcycling of polyethylene terephthalate. Metab. Eng..

[120] Furukawa M., Kawakami N., Tomizawa A., Miyamoto K. (2019). Efficient Degradation of Poly(ethylene terephthalate) with Thermobifida fusca Cutinase Exhibiting Improved Catalytic Activity Generated using Mutagenesis and Additive-based Approaches. Sci. Rep..

[121] Gu J.D. (2021). Biodegradability of plastics: The issues, recent advances, and future perspectives. Env. Sci. Pollut. Res. Int..

