# Supplementary material for: Current Approaches to Microplastics Detection and Plastic Biodegradation
Source: Molecules. 2025 Jun 4;30(11):2462. doi: 10.3390/molecules30112462 (PMC12156231; doi:10.3390/molecules30112462)
Supplement: Supplementary file 1 [file molecules-30-02462-s001.zip › molecules-3631387-supplementary.pdf]

molecules

MDPI

Review

Current approaches to microplastic detection and plastic biodegradation

Paula Przygoda-Kuś [1], Katarzyna E. Kosiorowska[1,2], Aneta K. Urbanek[1] and Aleksandra M. Mirończuk [1,*]

[1] Wrocław University of Environmental and Life Sciences, Institute of Biology, Laboratory for Biosustainability, Kożuchowska 5b, 51-631 Wrocław, Poland

[2] Wroclaw University of Environmental and Life Sciences, Department of Applied Bioeconomy, Chełmońskiego Str. 37a, 51–630 Wrocław, Poland

* Correspondence aleksandra.mironczuk@upwr.edu.pl

Supplementary Materials

Table S1.A Comparison of methods for identification and characterization of microplastics with examples of applications and advantages, and limitations of these methods.

Detection methods	Plastic Type	Matrix	Detection parameters	Advantages	Limitations	References
**Microscopic methods**						
**SEM**	LDPE, HDPE, PP, PVC	bacterial culture	- Zeiss EVO 18 equipped with secondary electron detector with an accelerating voltage of 200 V-30 kV -plastic films plated with gold particles	- high-quality images - small detected particles	- sample preparation and inspection require significant time and effort - lack of information on type of polymer	[23, 25]
**Fluorescence microscopy**	LDPE, HDPE, PE, PET,	beach sand, sea water	- three excitation and emission wavelengths: blue (excitation wavelength (ex.): 365 nm; emission wavelength (em.): 445 nm), green-	- accessible - immediate visualisation of particles	- chemical additives can interfere with fluorescence	[23, 38-40]

	PP, PVC, PU		yellow (ex.: 450–490; em: 515–565 nm), and orange- red (ex.: 534–558; em.: N 590 nm)<br>- Zeiss Axio Scope A1	- time and cost-effective		
**TEM**	PET	fish tissue, washing water, bacterial cultures, medusae tissue, daphnia tissue	-transmission electron microscope JEOL JEM-1400 Plus at 5000–10000 × magnification	-high resolution for nanoscale samples<br>-excellent image quality and magnification	-elevated price<br>-possible masking of smaller particles by larger ones<br>-representativeness of the acquired data might not be sufficient, especially for heterogeneous samples<br>-sample preparation and inspection require significant time and effort<br>-the need for ultrahigh vacuum environment	[27, 28, 101]<br>[30, 102-104]
	PET, PP		-staining of TEM grids with commercially available UranyLess (Electron Microscopy Sciences) and lead citrate (Electron Microscopy Sciences) solutions for improving the contrast<br>-Tecnai Spirit transmission electron microscope (FEI) operating at 120 kV with a wide angle Veleta CCD camera (2048 × 2048 pixel; Olympus)			
	PS beads		-TEM (JEM-2100, JEO, Japan)			
	HDPE		-EOL JEM-1400 PLUS microscope at 100 kV (JEOL Ltd., Japan) using TEM Centre for JEM1400 Plus software<br>-samples were placed onto a pioloform-coated single slot grid (Ted Pella, Cu, Pelco Slot Grids, USA)			

Table S1.B. Comparison of methods for identification and characterization of microplastics with examples of applications and advantages and limitations of these methods

**Detection methods**	**Plastic Type**	**Matrix**	**Detection parameters**	**Advantages**	**Limitations**	**References**
**Spectroscopic methods**						
**Raman Spectroscopy**	PET	commercial polymers, seawater, freshwater, wastewater, sediment, food, drink, cosmetics, aquatic organisms' tissues, soil	Automated scanning of sub-sections, Int. time 0,02 s, laser power 2.05 mW, working distance 3 mm,	- high sensitivity detection,<br>-detailed detection method including information about the structure of molecules,<br>- does not cause mechanical damage to samples,<br>- rapid analysis time,<br>- possibility of analyzing small sample sizes,<br>- enables detection of various types of polymers,<br>- low cost of analysis.	- small sample size may limit sensitivity,<br>- requires careful sample preparation to minimize contamination,<br>- some polymers may have similar spectral properties<br>- water interference can affect results: water signal can overshadow or interfere with those of MPs,<br>- interpretation of spectra may require specialized knowledge<br>- limited information on morphology of samples.	[42, 44]
	PE					
	PP					
	PS					
**XPS**	PE, HDPE	mussels' tissues,	-samples were vacuum dried for at least 1 week before use	-nondestructive for many materials	-limited insights into the bulk composition of MPs,	[25, 50, 52, 53, 103, 105]

		radish seeds, tea, tap water with polishing dust, commercial polymers, bacterial cultures	-VG 220i-XL equipped with a monochromated Al K-alpha X-ray source<br>-data analysis was done using the CasaXPS software (version 2.3.18)	-good for analyzing thin films<br>-depth profiling of the elemental composition<br>-effective at identifying surface contaminants<br>-analysis can be done in as little as 30 minutes<br>-sensitive enough to detect variations in the degree of oxidation	the average depth of analysis is approx. 5 nm<br>-meticulous sample preparation<br>-potential false positives or negatives signals<br>-cannot be used for single-particle analysis but rather for collection measurements<br>-the sample cannot exceed 25 mm in any lateral direction, height should not exceed 12 mm<br>-10% relative error in repeated analyses<br>-samples must be amenable to a high vacuum environment	
	PET, nylon		-Kα X-ray photoelectron spectrometer, using a monochromatic Al Kα X-ray source and a flood gun in a 10^{-8} mbar vacuum (Thermo Scientific)			
	LLDPE, HDPE, PBAT, PP, PVC		-collection of the spectra at a constant passage energy mode of 100 eV, calibration of all binding energies using a 284.8 C1s peak			
**XRD**	PP, PE, PET, PE, PVC	commercial polymers, mealworm larvae tissues, soil, marine water	-Empyrean diffractometer (Malvern Panalytical, UK) in reflection mode with a primary beam monochromator (reflection mode Cu Kα1)	-nondestructive method<br>-minimal sample quantities with optimum size less than 10µm<br>-simple and most effective way to	-changes in polymer crystallinity do not always correspond to the rate of biodegradation<br>-can be time-consuming<br>-low intensity of diffracted X-rays, specifically for low atomic number material	[54, 56, 106, 107]
	PS, PVC, LDPE		-Bruker D8 Advance diffractometer operated at 40 kV and 40 mA with Cu-Ka radiation (k = 1.5406 A) and a			

			diffracted beam monochromator, using a step scan mode with the step size of 0.075° (2h) and scan rate of 1.2°/min	quantify polymer degradation by calculating the change from crystalline to amorphous nature<br>-provide information on the molecular and chemical structure of the surface region<br>-can be used to analyze a wide variety of materials, including solids, powders, and liquids		
	PET, PE, PP, PS, PBT		-the spectra obtained by Bruker model D8 (Germany)<br>-analysis performed in the range 1° to 80° under 2Θ diffraction angle			
	PET, PCL, PVC, LLDPE, PS, PP		-Empyrean XRD instrument (Malvern Panalytical, Netherlands), operated with CuKα1 and Kα2 radiations at the wavelength of λ = 1.5405 Å and 1.5444 Å<br>-copper radiation produced using a generator voltage of 40 kV and tube current 40 mA<br>-scanning of samples between a 2θ range of 10° to 80° with a step size of 0.006 and time per step of 80 s			
**FTIR**	PET	waste plastics, commercial polymers, microorganisms' cultures,	TG-FTIR analysis, wavelengths range: 4000-600cm^{-1}, transmission and cell temperature 250°C, data collection at sample temperature 300°C	- applicable to various types of polymers,<br>- can be used for qualitative analysis,<br>- does not cause damage to samples during analysis,	- the application of the method is limited to the surface of the sample, subsurface structures are not detectable,<br>- the limit of detection and accuracy depends on the	[108]
			Wavelengths range: 4000-500cm^{-1}			[109]

			ATR-FTIR analysis, wavelength range 1800-500 cm^{-1}, resolution 4 cm^{-1}, 4 scans	- ensures fast analysis time, which enables examination of a large number of samples in a short period,<br>- possibility to use the method for samples of different origin,<br>- simplicity of equipment usage.	size of the sample, which may limit its application for the analysis of smaller particles,<br>- in environmental samples, precise identification may be impaired due to the presence of other compounds,<br>- the risk of overlapping spectra, which can be problematic for environmental samples, complicating accurate polymer identification,<br><br>- for MPs analysis, concentration or purification of samples of environmental origin may be required,	[110]
	PP		Wavelength range 4000-650 cm^{-1}, resolution 4 cm^{-1}, 16 acquisitions (co-added scans)			[59]
	HDPE/LDPE		Wavelength range 4000-500 cm^{-1}, resolution of 1 cm^{-1}			[111]
	PS		TG-FTIR analysis, wavelengths range: 4000-600cm^{-1}, transmission and cell temperature 250°C, data collection at sample temperature 300°C			[108]
	PUR		TG-FTIR-MS analysis, wavelength range 4500-600cm^{-1}, sample analysis temperature 800°C, heating rate 10°C/min, transmission temperature 190°C, gas used: helium, gas flow rate 75 $mLmin^{-1}$			[112]
			Wavelength range 4000-650cm^{-1}, resolution 4 cm^{-1} for 8 scans			[113]
			Wavelength range 3500-500cm^{-1}			[114]

	PVC		TG-FTIR analysis, wavelength 4000–450 cm^{-1}, 8 scans, cell temperature 280℃, gas used: nitrogen, gas flow rate 50 $mLmin^{-1}$, TG temperature range: 30-800 ℃			[115]
**NTA**	PS	commercial polymers, washing water, tea, daphnia tissues	-the sample chambre equipped with a 640-nm laser and a Viton fluoroelastomer O-ring<br>-the samples were injected in the chamber with sterile syringes until the liquid reached the tip of the nozzle<br>-room temperature	-sample visualization<br>-easy detection of contaminants<br>-accurate for both monodisperse and polydisperse samples	-up to 1 hour per measurement<br>-requires several optimization steps by a skilled operator<br>-applicable for a relatively narrow range of particle size	[50, 67, 101]
	PET		-analyzer Nanosight LM20<br>-solution introduced to the viewing cell, and 60s videos were recorded			
	PET, nylon		-LM14 instrument with a 532 nm green laser, NanoSight Ltd.			
	HDPE		-NanoSight LM10 (Amesbury, UK) instrument<br>-analysed with software NanoSight NTA 3.1 with a standard analysis setting			

**DLS**	PS	commercial polymers, microorganisms' cultures, daphnia tissues	-Malvern Zetasizer Nano ZS equipped with a 633-nm He-Ne laser and operating at an angle of 173° -measured in single-use polystyrene half-micro cuvettes with a pathlength of 10 mm	-between 2 to 5 min per measurement -little sample handling and user-friendly -reliable and repeatable analysis -applicable for a relatively broad range of particle sizes	-large particles can seriously compromise the results -hard detection of contaminants -inaccurate for polydisperse samples -not capable to measure particle concentration -highly sensitive to temperature and solvent viscosity	[27, 67, 102, 116]
	HDPE, LDPE		-SALD-7500nano particle analyzer (Shimadzu, Japan) -analysis performed using WingSALD II version 3.1.1			
	HDPE		-DynaPro Plate Reader II, Wyatt Technology Corp, USA -samples were recorded at 23 °C for 10 s 10 times -data was analysed using the Dynamics V7 program			

Table S1.C. Comparison of methods for identification and characterization of microplastics with examples of applications and advantages and limitations of these methods

Detection methods	Plastic Type	Matrix	Detection parameters	Advantages	Limitations	References
**Chemical and analytical methods**						
**Weight loss**	PET, PP, PE, PS and others	microorganisms' cultures,	-weight (mg, g)	-quick,<br>-simply<br>-low costs<br>-does not require an expensive instruments	-inaccuracy,<br>- human measurement error	[4, 24, 25, 70]
**Clear zone**	PET, PCL, PBSA	microorganisms' cultures,	-diameter of the zone (mm)	- quick and cheap technique,<br>-good and useful for screening,<br>-does not require a qualified staff	-inaccuracy,<br>-emulsions are unusual materials,<br>-human measurement error	[78, 79]
**CA**	LLDPE, HDPE, PBAT, PP, PVC	microorganisms' culture, soil	-measured at room temperature	-fast and easy to perform<br>-inexpensive	-not accurate for very hydrophobic surfaces<br>-surface chemistry information is ambiguous	[25, 74, 82]

	LDPE		-water angle contact detector (JC 2000D1; Powereach, China)		-liquid purity is of critical importance	
	PET, PS		-precision contact angle tensiometer (DCAT 11, DataPhysics, Filderstadt, Germany)			
**TGA**	PS, PVC, LDPE	mealworm larvae tissues, marine water	-thermogravimetric analyzer TGA-50 (Shimadzu, Japan)<br>-heating rate of 10 °C/min from ambient temperature to 600 °C under high-purity nitrogen (99.99%) at a flow rate of 40 mL/min	-low-cost technique<br>-needs a small sample<br>-allows for quantitative or qualitative analysis	-destructive technique<br>-not useful for plastics with a high amount of additives<br>-not give the exact identification of the gasses produced from the sample during heating<br>-might not to be precise enough due to the presence of volatile chemicals in the sample	[103, 106, 107]
	PET, PCL, PVC, LLDPE, PS, PP		-Perkin Elmer Thermogravimetric Instrument (STA-8000)<br>-samples loaded in an alumina crucible and maintained under an N_2 atmosphere with a flow rate of 50 mL min^{-1} and a temperature gradient from 30 °C			

			to 800 °C, with a heating rate of 5 °C min^{-1}			
**DSC**	PP, PE, PET, PE, PVC	commercial polymers, marine water, sand, withered leaf, soil, sludge water	-DSC250 (TA, USA) with N_2 as the purge gas<br>-heating-cooling-heating method was applied	-low sample amount<br>-low cost<br>-liquid and solid availability for analysis<br>-identification of various physical properties and thermal transitions of polymeric materials such as the glass transition temperature (T_g), the melting temperature (T_m) and the enthalpy change associated to T_m (ΔH_m)	-destructive technique<br>-analyzing heterogeneous samples might be challenging<br>-basing on the thermal properties of the entire sample mass and therefore is less likely to reflect surface changes	[54, 89, 103, 106]
	PET, PE, PP		-Netzsch DSC 214 Polyma (Netzsch-Gerätebau GmbH, Selb, Germany) with nitrogen as purge and protective gas<br>-heating (303.2 to 563.2 K at 0.333 Ks^{-1}) - cooling (563.2 to 273.2 K at 0.167 Ks^{-1}) - heating (273.2 to 563.2 K at 0.083 Ks^{-1}) method was applied			
**Pyr-GC/MS**	PS, PC, PP,PE, PET, PVC, PMMA, N-6, N-66, PUR,	commer-cial poly-mers, beach sand, bivalves' tissues, sea surface waters	-multi-Shot EGA/PY-3030D micro-furnace pyrolyzer and an Auto-Shot sampler AS-1020E ,Frontier Laboratories Ltd.	- high sensitivity<br>-the identification and quantification of MPs<br>-requires small amount of the samples	-require Pyr-GC/MS<br>-require experienced staff for analysis	[92, 95]

	ABS, SBR					
**UPLC**	PET	microorganisms' cultures, enzyme-containing medium	Column: Hypersil GOLD™ C18 (100 × 2.1 mm, 3 µm). Mobile phases: acetonitrile with 0.1% trifluoroacetic acid and H2O with 0.1% trifluoroacetic acid (gradient elution). Elution time: 7 min. Flow rate: 0.8 mLmin^{-1}. Injection volume: 1 µL. Column temperature: 45 °C	-the method allows rapid sample analysis. -the price of the column is relatively low. The eluents used are commonly used in analytical chemistry.	Sample preparation time requires double centrifugation at 10 °C. It is suggested to use an autosampler with cooling. This method is applicable to the detection of degradation products such as TPA, MHET and BHET. For EG, additional analysis using a different column (e.g., Carbohydrate H+) is necessary.	[21, 117]
			Column: Tosoh TSK-GEL2500PW. Mobile phase: 0.3 M $NaNO_3$ (isocratic elution). Elution time: 20 min. Flow rate: 1.0 mLmin^{-1}. Injection volume: 50 µL. Column temperature: 30 °C	High separation efficiency.	Relatively long analysis time (20 min). The column used is mainly aimed at the analysis of water-soluble linear polymers with a maximum mass of 3000 Da.	[118]
			Column: C18 Eurospher 100-5, 150 mm × 4.6 mm.	According to the described method	Described method is suited only for TPA,	[119]

			Mobile phase: 20% acetonitrile, 20% 10 mM sulfuric acid 60% Mili-Q water. Elution time: N/A. Flow rate: 1 mLmin^{-1}. Injection volume: 10 μL. Column temperature: N/A	characteristic, it may be assumed that analysis time is relatively fast. The eluents used are commonly used in analytical chemistry.	MHET and BHET quantification. EG has to be analyzed with the use of different column and conditions.	
			Column: Cosmosil 5C18 -AR-II. Mobile phase: 70% Milli-Q water, 20% acetonitrile, 10% formic acid. Elution time: 7 min. Flow rate: 1 mLmin^{-1}. Injection volume: 10 μL. Column temperature: 40 °C	Rapid analysis time. Commonly used chemicals as eluents.	Described method is suited only for TPA, MHET and BHET quantification. EG has to be analyzed with the use of different column.	[120]
	PP		Columns: C18 type columns (Zorbax ODS1, ZORBAX Eclipse Plus C18, BEH 18, Discovery C18) Mobile phase: 50% (v/v) methanol in water Elution time: n/a Flow rate: 0.5 mLmin^{-1} Injection volume: n/a Column temperature: 40 °C	Quick analysis, commonly used column and chemical as eluent.	This method enables analysis of 2-phenyl ethanol and styrene. To analyze the whole scope of degradation products it is necessary to use several columns enabling separation of specific compounds- there is no single method allowing separation of all possible analytes.	[121]
